# Association between Health-Related Quality of Life and Medication Adherence in Pulmonary Tuberculosis in South Africa

**DOI:** 10.3389/fphar.2017.00919

**Published:** 2017-12-18

**Authors:** Tanja Kastien-Hilka, Bernd Rosenkranz, Matthias Schwenkglenks, Bryan M. Bennett, Edina Sinanovic

**Affiliations:** ^1^Epidemiology and Public Health Department, Swiss Tropical and Public Health Institute, Basel, Switzerland; ^2^Department of Public Health, Faculty of Medicine, University of Basel, Basel, Switzerland; ^3^Health Economics Unit, School of Public Health and Family Medicine, University of Cape Town, Cape Town, South Africa; ^4^Division of Clinical Pharmacology, Faculty of Medicine and Health Sciences, Stellenbosch University, Cape Town, South Africa; ^5^Fundisa African Academy of Medicines Development, Cape Town, South Africa; ^6^Institute of Pharmaceutical Medicine, University of Basel, Basel, Switzerland; ^7^Epidemiology, Biostatistics and Prevention Institute, University of Zürich, Zurich, Switzerland; ^8^Patient Centered Outcomes, Adelphi Values, Bollington, United Kingdom

**Keywords:** quality of life, pulmonary tuberculosis, medication adherence, longitudinal study, patient-reported outcomes

## Abstract

**Background:** Health-related quality of life (HRQOL) and adherence to treatment are two often inter-related concepts that have implications for patient management and care. Tuberculosis (TB) and its treatment present a major public health concern in South Africa. The study aimed to evaluate the association between HRQOL and adherence in TB patients in South Africa.

**Methods:** Four self-reported HRQOL and one self-reported adherence measures were used in an observational longitudinal multicentre study during 6-month standard TB treatment. These included the generic Short-Form 12 items (SF-12) and European Quality of Life 5 dimensions 5 levels (EQ-5D-5L), the disease-specific St. George's Respiratory Questionnaire (SGRQ) and the condition-specific Hospital Anxiety and Depression Scale (HADS) for HRQOL. Adherence was measured by the Morisky Medication Adherence Scale 8 items (MMAS-8). The relationship between both concepts was examined in 131 patients using Spearman's rho correlations, and linear regression models.

**Results:** HRQOL improved over 6-month TB treatment, whereas adherence mean scores stayed constant with participants attaining a medium average level. Around 76% of patients reported to be high adherers and 24% were reporting a medium or low adherence. Associations between HRQOL and adherence were mainly weak. High adherence at treatment start was positively related to improvements in anxiety and depression after 6-month treatment. The overall improvement in pain and discomfort, and psychosocial health aspects over treatment time was positively, but weakly associated with adherence at 6 months of treatment.

**Conclusion:** A positive relationship exists between adherence and HRQOL in TB in a South African setting, but this relationship was very weak, most likely because HRQOL is affected by a number of different factors and not limited to effects of adherence. Therefore, management of TB patients should, besides adequate drug treatment, address the specific mental and psychosocial needs.

## Introduction

Both adherence and health-related quality of life (HRQOL) are important indicators for the effectiveness and treatment success of therapeutic interventions (Côté et al., 2003). Determinants of adherence behavior are well-established in the literature (Kardas et al., 2013). Psychosocial factors, such as poor levels of education, have been shown to influence whether patients are adherent with their medication. Treatment-related factors, such as complexity (Pantuzza et al., 2017) and number of drugs (Sabaté, 2003), also have a negative impact on adherence in many different chronic conditions. It is expected that poor adherence leads to poorer outcomes, including HRQOL, because if patients do not take their prescribed medication, then it will have no therapeutic effect. Treatment of tuberculosis (TB) is generally in two phases. The first phase is an intensive phase typically combining four drugs during 2 months. The second phase, involving two drugs for 4 months, is often termed a continuous phase of treatment. The complexity of TB treatment regime is a consideration for adherence.

However, the association between HRQOL and adherence to treatment has rarely been studied in tuberculosis (TB). Cote et al. and Saleem et al. propose a model whereby HRQOL is an ultimate outcome while adherence is seen as an intermediate outcome (process variable). This model assumes that adherence to therapeutic interventions drives changes in HRQOL occurring with a delay (Côté et al., 2003; Saleem et al., 2012). However, the relationship between the two concepts is complicated by a possibility of reverse causality, i.e., a “backwards” influence of HRQOL on adherence (Ágh et al., 2015). A systematic review by Munro et al. identified two underlying mechanisms for the association between well-being and adherence, specific to TB treatment (Munro et al., 2007). The first mechanism relates to TB patients prematurely stopping their treatment because they feel better, and perceive the improvement in well-being as a cure of TB. On the other hand, the second observed mechanism refers to patients stopping treatment when they experience no improvement or a worsening in their health status and well-being, because they perceive the medication as not working (Sabaté, 2003; Munro et al., 2007). This alternative model implies that a change in HRQOL would appear first, and be followed by a change in adherence behavior.

South Africa, which has the highest prevalence and incidence rates of all 22 high-burden TB countries worldwide (WHO, 2016; Wang et al., 2017), has committed a significant portion of its health budget to expand early detection and treatment of TB (Christian, 2016; Kastien-Hilka et al., 2016; Dieleman et al., 2017a; Jakovljevic et al., 2017). Aside from the financial burden on the South African health budget, TB also places a considerable burden on patients, significantly impacting the physical, emotional, psycho-social and economic domains of health (Dieleman et al., 2017b). Whilst available free of charge in the public sector, a major challenge faced by the National TB Programme and patients is the length and complexity of existing treatment regimens. The incidence rate is slowly decreasing and the relapse rate is high (WHO, 2016). These same domains are known to impact medication adherence (Sabaté, 2003). The aim of this research is to evaluate the relationship between patient-reported HRQOL and adherence in pulmonary TB in South Africa using a longitudinal study design. The evaluation of both concepts over time allows identifying which health aspects of TB have an influence on adherence and which health domains are affected by non-adherence.

## Materials and methods

### Study design

The study used an observational longitudinal design including repeated measures of HRQOL and adherence. A detailed description of the study design and methodology has previously been published and briefly presented below (Kastien-Hilka et al., 2016b).

### Patient population and participant recruitment

Study participants were recruited between November 2014 and May 2015 at six primary health care clinics with the highest TB caseloads in Cape Town. The study population comprised of 131 patients newly diagnosed with active pulmonary TB who were 18 years of age or older. A detailed description of the study population has been published elsewhere (Kastien-Hilka et al., 2017). Of those who provided socio-demographic data (*n* = 129), 63.6% were man, 89.9% were black, the majority were between 20 and 40 years of age (46.5%), and 68.3% had high school attainment. Just 26.6% of all participants reported currently being employed or doing any kind of paid work.

Patients were excluded if they were diagnosed with multidrug-resistant TB (MDR-TB), extensively drug-resistant TB (XDR-TB), and/or had HIV co-infection. The eligibility status of each patient was subject to verification by the nurse dedicated to TB patients at each health facility. All enrolled participants signed a written informed consent.

### Study procedures

Self-reported HRQOL data was collected at the beginning of the 6-month treatment period and at four follow-up visits (4, 8, 16 weeks and at the end of treatment) at each of the sites included in the research. Four HRQOL measures and one socio-demographic questionnaire were administered at baseline (treatment start). These four HRQOL measures were administered again, together with a self-reported adherence measure at the follow-up visits. Adherence to medication data was collected during the follow-up visits only, as no treatment was administered until after baseline data were collected. Data were collected using paper questionnaires during face-to-face interviews conducted by trained field workers.

### Study material

The rationale for the selection of HRQOL measures and adherence measure has been described previously (Kastien-Hilka et al., 2016b). The self-reported questionnaires consisted of; two generic quality of life questionnaires [Short-Form 12 items (SF-12; Jenkinson et al., 1997) and European Quality of Life 5 dimensions 5 levels (EQ-5D-5L; Herdman et al., 2011)], one disease-specific quality of life questionnaire [St. George's Respiratory Questionnaire (SGRQ; Jones et al., 1992)] and one condition-specific questionnaire [Hospital Anxiety and Depression Scale (HADS; Zigmond and Snaith, 1983)]. Adherence to medication was measured using the Morisky Medication Adherence Scale 8 items (MMAS-8; Morisky et al., 2008; Krousel-Wood et al., 2009; Morisky and DiMatteo, 2011).

### Statistical analysis

All statistical analyses were conducted using IBM SPSS Version 23®. Descriptive analyses were applied to baseline socio-demographic and adherence data by follow-up visit (N, % missing, min, max, mean, *SD*, median and 95% Cis). Frequency plots were also produced. Adherence data were also assessed by levels of adherence (high adherence = 8; medium adherence = 6– <8; low adherence <6).

Longitudinal changes in adherence were examined by the change in mean scores between follow-up visits. The change in mean scores was assessed by paired-samples *t*-test at a 5% significance level. Differences in adherence mean scores over time were also examined using repeated measures ANOVA. Bonferroni correction was applied to account for multiple testing. The effect size partial eta squared (η^2^) was used to provide information of the within-subject effect of time. Adjustments were made for baseline level socio-demographic factors.

Associations between HRQOL and adherence mean scores were examined using a step-wise method. First, relationships between descriptive HRQOL and adherence measures were explored. Second, Spearman's correlation coefficients were calculated between HRQOL domain-level and total mean scores and adherence mean scores at each follow-up visit. Further, Spearman's correlation coefficients were examined between changes in HRQOL mean scores (between baseline and both follow-up visit 2 and follow-up visit 4) and adherence mean scores at follow-up visit 4. Correlations >0.5 will be considered as strong, between 0.3 and 0.5 as moderate, 0.1 and 0.3 as weak and <0.1 as no correlation (Cohen, 1988). In a third step, linear regression models were used to examine the associations over time. Domain-based associations identified from the correlation analysis were examined for confirmation.

### Ethical consideration

The study adhered to the International Conference on Harmonisation of Technical Requirements for Registration of Pharmaceuticals for Human Use (ICH) guidelines, the Declaration of Helsinki and South African Good Clinical Practice (GCP). The study was approved by the institutional review commission of the Swiss Tropical and Public Health Institute in Basel, the ethics committees of North-West and Central Switzerland (EKNZ) and the University of Cape Town.

## Results

### Development of HRQOL over time

Results from the self-reported HRQOL questionnaires revealed significant impairment in overall HRQOL and domains at baseline. Analyses identified socio-demographic determinants associated with baseline HRQOL were age, education and work status; where older, less educated and unemployed patients report poorer HRQOL. Gender showed no significant effect on HRQOL. HRQOL total and domain scores improved over 6-month of TB treatment, with greatest improvement observed during the intensive treatment phase. The changes in HRQOL were statistically significant, and exceeded the published thresholds for minimal important difference (MID) indicating the differences can be interpreted as clinically meaningful. The greatest improvement in HRQOL was observed in mental health domain, such as anxiety and depression. Details have been published recently (Kastien-Hilka et al., 2017).

### Development of adherence over treatment time

**Figure 1:**
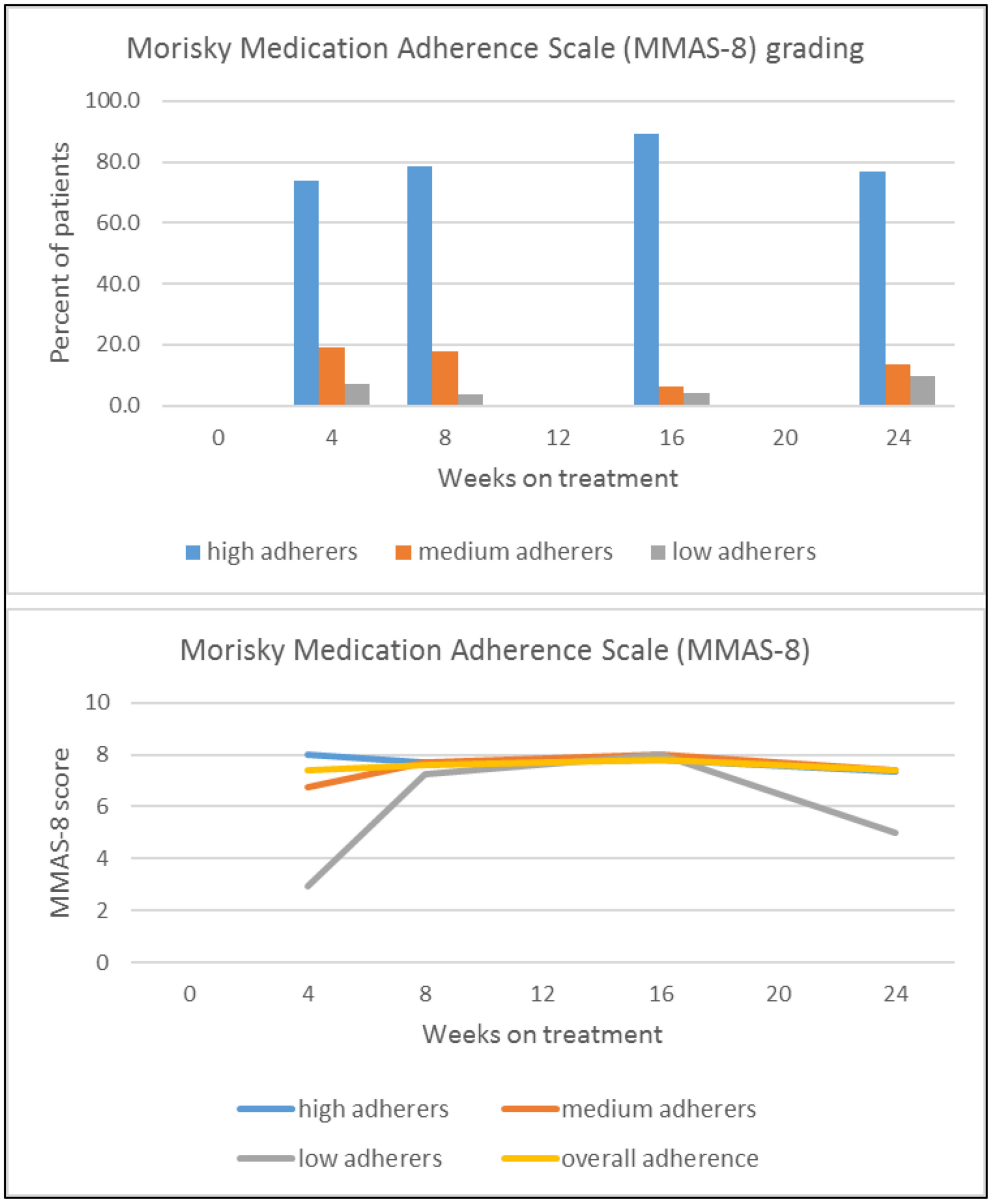
Absolute proportion of high, medium and low adherers **(Top)**, and change of adherence over time, by adherence level at week 4, visit 1 **(Bottom)**.

Table 1 and Figure 1 present self-reported adherence from 4 weeks (follow-up visit 1) to 6 months (follow-up visit 4) of standard TB treatment. The mean MMAS-8 score for all participants was in the range of medium adherence over all time points. Based on the adherence grading of MMAS-8, the majority of TB patients reported high adherence at all follow-up visits during treatment (74–89%), as shown in the top panel of Figure 1. This high level of adherence remained unchanged in most patients (79%) throughout the study period. However, ~21% of patients classified as high adherers at follow-up visit 1 changed their adherence behavior to medium and low adherence after 6 months (at follow-up visit 4). In the group of patients with medium and low adherence, 46% improved their adherence to high adherence behavior after 6 months of treatment. Adherence at week 4 (follow-up visit 1) did not appear to be predictive of adherence at later time points, as visible from Table 1 and Figure 1 (bottom panel).

**Table 1:** Change in adherence over time (measured using the MMAS-8) by adherence level at first visit (Jenkinson et al., 1997)^a^.

**Time since treatment start**	**Mean change from Visit 1, N**	**95% confidence interval, Range**	**High adherers at first visit (74%) N^b^**	**Medium adherers at first visit (19%), N^b^**	**Low adherers at first visit (7%), N^b^**
Week 4 (visit 1) (mean, *SD* [%])	7.400 (1.474) 84	(7.076-7.716)	8.000 62	6.734 15	2.917 6
Week 8 (visit 2)	+0.222 84	(7.394-7.851)	−0.283 46	+0.991 10	+4.333 3
Week 16 (visit 3)	+0.400 47	(7.601-7.995)	−0.205 28	+1.266 5	+5.083 1
Week 24 (visit 4)	+0.400 95	(7.089-7.701)	−0.659 52	+0.691 10	+2.083 2

a *MMAS-8 scores were not obtained during baseline (N = 131) as treatment had not commenced*.

b *Adherence was classified as “High” (MMAS-8 score = 8), “Medium” (MASS-8 score = 6- < 8) or “Low” (MASS-8 score < 6)*.

*Use of the ©MMAS is protected by US copyright laws. Permission for use is required. A license agreement is available from Donald E. Morisky, 14725 NE 20th St Bellevue, WA 98007; dmorisky@gmail.com*.

*MMAS-8, Morisky Medication Adherence Scale 8 items; SD, standard deviation*.

Age, gender and work status revealed no impact on adherence at follow-up visits 1 and 4. Only education had a statistically significant influence on the adherence at follow-up visit 4 (η^2^ = 0.129, *P* = 0.016). Specifically, at 6 months of treatment a higher educational background was associated with lower adherence scores. In a multivariate regression model retaining non-significant effects of age, gender and work status, the MMAS-8 score in persons with high school education was by 0.25 (*P* = 0.541) lower than in persons with primary school education. It was by 1.71 (*P* = 0.011) lower in persons with college or university education (*P* for the set of dummy variables representing education, 0.027; value range of MMAS-8, 0-8).

### Association between HRQOL and adherence over treatment time

**Figure 2:**
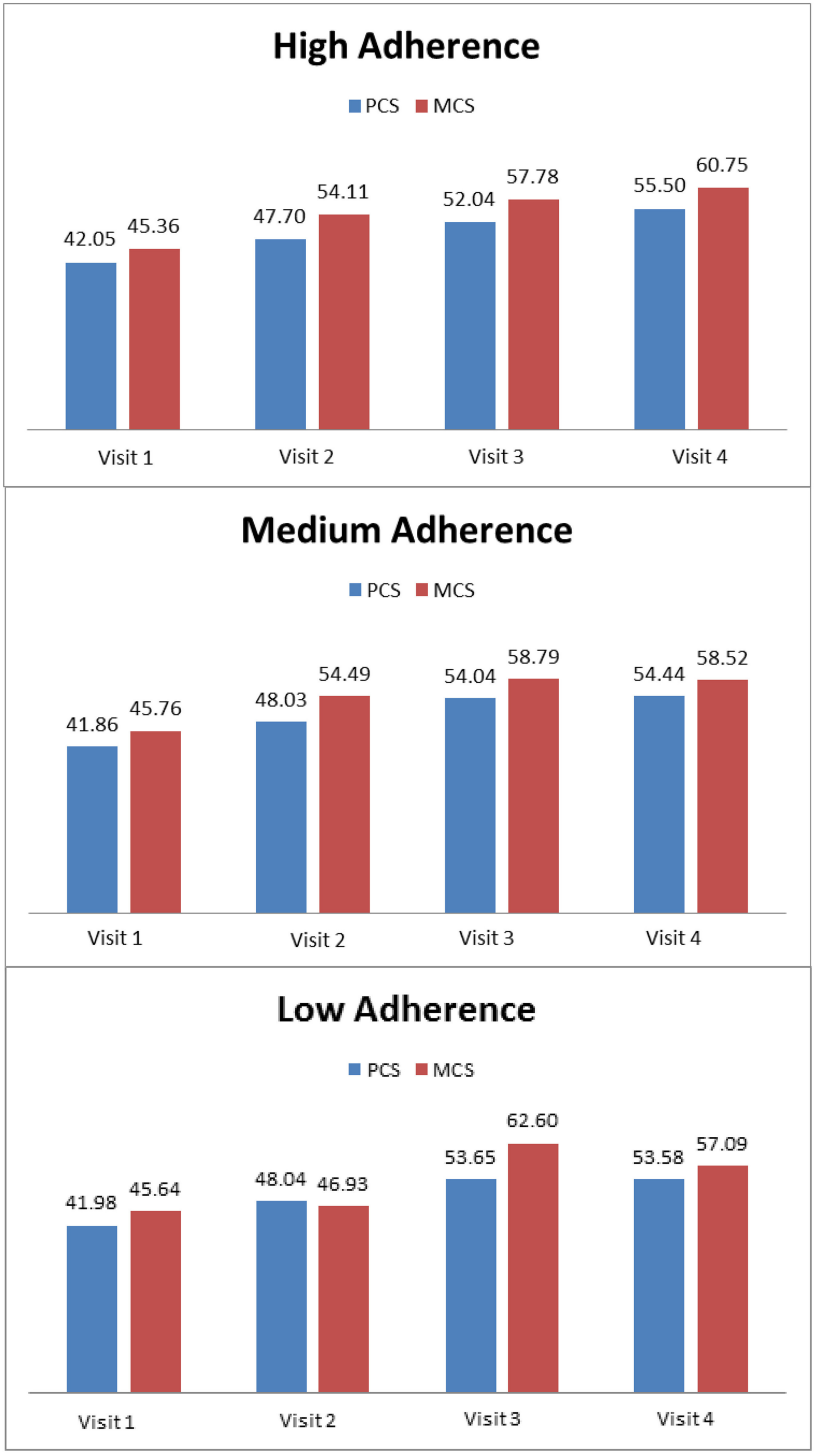
Association between HRQOL and adherence (high, medium or low) over all follow-up visits using composite summary scores, Physical Component Score (PCS), and Mental Component Score (MCS) of the SF-12.

Figure 2 presents the association between HRQOL and level of adherence (high, medium, or low), over all follow-up visits. HRQOL improved significantly over the treatment time, while overall mean adherence scores remained constant at a medium adherence level. The most pronounced correlation identified indicated a moderate and positive association between adherence in the first 4 weeks (follow-up visit 1) and less problems in the Anxiety/Depression domain of the EQ-5D after 6 months of treatment (follow-up visit 4; *r* = 0.430 P < 0.001). A similar positive but weak relationship was observed between adherence in the first 4 weeks (follow-up visit 1) and EQ-5D utility index after 6 months of treatment (follow-up visit 1; *r* = 0.364 *P* = 0.003). After 6 months of treatment (follow-up visit 4), a weak but positive relationship between the SGRQ Symptoms and Activities domains and adherence was observed (*r* = −0.297 *P* = 0.004; *r* = −0.317 *P* = 0.002).

Spearman's correlation was also calculated for overall changes in HRQOL mean scores (baseline to follow-up visit 4) and adherence after 6 months of treatment. Statistically significant, but weak correlations were observed with the overall improvement of the HADS Anxiety (*r* = 0.226 *P* = 0.026), EQ-5D Pain/Discomfort (*r* = 0.232 *P* = 0.026) and SGRQ Impacts (*r* = 0.291 *P* = 0.005) domains, and with the SGRQ total score (*r* = 0.226 *P* = 0.014). All these results indicated that an improvement in HRQOL between baseline and follow-up visit 4 was positively associated with higher adherence.

The multivariate model confirmed some of these findings. It showed statistically significant, but very weak, positive associations between improvements in the EQ-5D Pain/Discomfort domain (η^2^ = 0.098 *P* = 0.041), SGRQ Activities domain (η^2^ = 0.110 *P* = 0.026), SGRQ Impacts domain (η^2^ = 0.131 *P* = 0.011) and SGRQ total score (η^2^ = 0.135 *P* = 0.013), and adherence at follow-up visit 4. The associations between HRQOL domains and adherence were responsible for 10–14% of the variance in the HRQOL domains. In addition, an association between improvement of SGRQ total score (η^2^ = 0.102 *P* = 0.039) in the first 8 weeks of treatment (follow-up visit 2) and adherence at follow-up visit 4 was found; this association accounted for 11% in the variance in SGRQ total score. Patients with high adherence had a greater change in HRQOL than patients with medium and low adherence. For the SGRQ Activities and Impacts domains as well as for the SGRQ total score, a worsening was observed for medium adherers.

The positive associations between overall improvements in the EQ-5D Pain/Discomfort domain (η^2^ = 0.071 *P* = 0.038), SGRQ Impacts domain (η^2^ = 0.088 *P* = 0.015) and SGRQ total score (η^2^ = 0.077 *P* = 0.031), and adherence after 6 months of treatment (follow-up visit 4) from the multivariate model were further confirmed by statistically significant results from repeated measures ANOVA.

Whilst no significant relationships between HRQOL and adherence levels were found during the intensive treatment phase, the above-described associations were confirmed for the continuous treatment phase. During the continuous treatment phase, there were significant and positive associations between HRQOL and adherence, with an effect size of 10–15%, related to the EQ-5D Pain/Discomfort domain (η^2^ = 0.103, *P* = 0.032), EQ-5D VAS (η^2^ = 0.156 *P* = 0.004), and SGRQ Activities (η^2^ = 0.123, *P* = 0.015) and Impact domains (η^2^ = 0.124 *P* = 0.014). High adherers reported a greater change in HRQOL than low adherers. Medium adherers showed a worsening in HRQOL during the continuous treatment phase in the SGRQ Impacts and Activities domains (Table 2).

**Table 2:** Associations between HRQOL and adherence during the intensive and continuous treatment phases.

**HRQOL**	**Spearman's rho correlation (HRQOL and adherence at 6 months of treatment) *r* (*P*-value)**	**Spearman's rho correlation (changes in HRQOL; adherence at 6 months) *r* (*P*-value)**	**Univariate model for continuous treatment phase η^2^ (*P*-value)**	**Multivariate model (changes in HRQOL; adherence at 6 months) η^2^ (*P*-value)**	**Repeated measures ANOVA η^2^ (*P*-value)**
EQ-5D Pain/Discomfort		0.232 (*P* = 0.026)	0.103 (*P* = 0.032)	0.098 (*P* = 0.041)	0.071 (*P* = 0.038)
SGRQ Activities	−0.317 (*P* = 0.002)		0.123 (*P* = 0.015)	0.110 (*P* = 0.026)	
SGRQ Impacts		0.291 (*P* = 0.005)	0.124 (*P* = 0.014)	0.131 (*P* = 0.011)	0.088 (*P* = 0.015)

*Significance level, 0.05. HRQOL, health-related quality of life; ANOVA, analysis of variance; EQ-5D, European Quality of Life 5 dimensions 5 levels; SGRQ, St. George's Respiratory Questionnaire*.

## Discussion

Despite treatment complexity being a predictive factor for low adherence in other conditions, the results of this study demonstrate that adherence to TB treatment within South Africa was high, and remained high throughout both the intensive and continuous treatment phases. The study showed that HRQOL improved in all domains, and that adherence behavior was constant, with most patients being classified as high adherer's during the course of 6-month standard treatment. Amongst baseline characteristics, only education had a significant effect on adherence after 6 months of treatment, with patients with lower education reporting better adherence. These patients also had lower HRQoL at baseline (Kastien-Hilka et al., 2017). Associations between HRQOL and adherence were statistically significant, but mainly weak. Generally, there were positive associations between adherence during the first four weeks of treatment and good HRQOL after 6 months of treatment in terms of lower levels of anxiety and depression and overall HRQOL. This would suggest that higher levels of adherence may positively influence the improvements in psychological distress. Our study did not find associations between HRQOL at treatment start and adherence over treatment time. However, other studies reported that an impaired HRQOL at treatment start negatively impacted adherence in patients with chronic obstructive pulmonary disease (COPD; Ágh et al., 2015).

Psychological distress, including anxiety and depression, are reported to be prevalent in TB patients (Kastien-Hilka et al., 2017). Theron et al. assessed psychological distress in TB patients in Southern Africa and observed a significant relationship between higher levels of TB-related psychological distress and non-adherence (Theron et al., 2015). In our study, depression at treatment start did not appear to be a trigger for reduced adherence. Rather, high adherence to treatment appeared to improve anxiety and depression.

## Limitations

The study is not without limitations. Combined generic with disease- and condition specific HRQOL measures were administered to ensure that all possible, relevant HRQOL domains were captured. However, there is no specific TB HRQOL measure. Generic measures such as SF-12 and EQ-5D may not be sensitive enough to examine associations with adherence. The EQ-5D is a preference-based instrument designed to only react to substantial changes in overall HRQoL. Given this, it may not be surprising that, in the multivariable analysis, we could confirm associations between adherence and individual EQ-5D domains, but not the overall EQ-5D utility index. We used a self-reported measure of adherence that may have impacted on the reliability of the observed relationship between HRQOL and adherence. An ideal adherence measure would assess overall adherence to medication including initiation, persistence, implementation and discontinuation. However, such a measure does not exist (Fairman and Motheral, 2000; Nguyen et al., 2014).

We were not able to look at the relationship between each of the adherence groups (high, medium, and low) and clinical outcomes for each of the patients who participated in this study. Further research would be required to examine adherence levels and clinical outcomes, perhaps using the sputum smear microscopy test to assess levels of TB bacteria.

Finally, for unknown reasons, the response rate at the follow-up visit 3 (after 16 weeks of treatment) was very low (Table 1). However, only few analyses made use of the date for this time point, such that a substantial impact on the overall results appears unlikely. The groups of medium and low adherers at the first follow-up visit were very small and subsequent assessments (for follow-up visits 2–4) performed in these groups were additionally affected by missing values.

## Conclusions

Very few studies have evaluated the association between different HRQOL aspects and adherence to treatment. Gaining a better understanding of adherence-related issues is a crucial step toward improving health outcomes and lowering health care costs. This study adds to the growing evidence for treatment adherence in TB patient populations specific to South Africa. A positive relationship exists between adherence and HRQOL in TB in a South African setting, but this relationship was very weak, most likely because HRQOL is affected by a number of different factors and not limited to effects of adherence. Therefore, management of TB patients should, besides adequate drug treatment, address their specific mental and psychosocial needs. Such integrative patient-centered approach could result in an improvement in the quality of life of these patients.

## Author contributions

TK-H conceived the study and led the design of the study. TK-H undertook the analyses and wrote the first draft of the manuscript. BR, MS, BB, and ES contributed to the design of the study, provided guidance on the analysis, reviewed and provided comments on the manuscript. BR, MS, BB, and ES reviewed and provided comments on the manuscript. ES, MS. and BB made further revisions to the manuscript following comments from reviewers.

### Conflict of interest statement

The authors declare that the research was conducted in the absence of any commercial or financial relationships that could be construed as a potential conflict of interest. The handling editor is currently co-organizing a Research Topic with one of the authors BR, and confirms the absence of any other collaboration.
